# A review of invasive intracranial pressure monitoring following surgery for hypertensive cerebral hemorrhage

**DOI:** 10.3389/fneur.2023.1108722

**Published:** 2023-07-04

**Authors:** Fu Chen, Shukui Zhang, Bingzhen Li, Jin Zhang, Maoxin Ran, Bin Qi

**Affiliations:** ^1^Department of Neurosurgery, The First Hospital of Jilin University, Changchun, China; ^2^Department of Neurosurgery, Sichuan Provincial People’s Hospital, University of Electronic Science and Technology of China, Chengdu, China; ^3^Department of Hepatobiliary Surgery, Zhijin County People's Hospital, Bijie, China

**Keywords:** invasive intracranial pressure monitoring, intracranial pressure, hypertensive cerebral hemorrhage, occupying effect, perihematomal edema

## Abstract

Hypertensive cerebral hemorrhage, the most common prevalent of spontaneous cerebral hemorrhage, poses a significant threat to patient mortality and morbidity, while therapeutic options remain limited, making the disease a burden not only for patients’ families but also a major challenge for national healthcare systems. The elevation of intracranial pressure subsequent to hypertensive cerebral hemorrhage is a critical contributor to mortality. However, it often manifests before the onset of clinical symptoms, which are typically atypical, leading to delayed treatment and irreversible consequences for the patient. Hence, early detection of intracranial pressure variations can aid in timely, efficient, and precise treatment, reducing patient mortality. Invasive intracranial pressure monitoring enables real-time, accurate monitoring of intracranial pressure changes, providing clinicians with therapeutic guidance and overcoming the limitations of empirical treatment. This article aims to review the use of invasive intracranial pressure monitoring in postoperative hypertensive cerebral hemorrhage and hopes to contribute to clinical and scientific research.

## Introduction

Cerebral hemorrhage represents a significant acute disease with a high rate of mortality and disability rate, comprising 10–15% of all stroke subtypes (1, 2). Hypertensive cerebral hemorrhage, accounting for the majority of spontaneous cerebral hemorrhages, is frequently associated with a history of hypertension in up to 70% of cases (3, 4). After hypertension-induced rupture of a cerebral blood vessel, blood penetrates the brain parenchyma, resulting in hematoma formation. On the one hand, this is the main cause of brain injury, as the hematoma occupies and compresses surrounding healthy brain tissue, causing destruction. On the other hand, the formation and retraction of the hematoma, as well as the extravasation of plasma proteins, can cause cerebral edema around the hematoma, which leads to a decrease in hydrostatic pressure around the hematoma space (5), The interaction between these factors can cause a pathological increase in intracranial pressure, ultimately leading to perihematomal edema (6, 7). Intracranial hypertension is common after cerebral hemorrhage, particularly in young and supratentorial cerebral hemorrhage patients (8). Intracranial hypertension is independently associated with patient clinical prognosis and mortality (9), Changes in intracranial pressure precede the onset of clinical signs and symptoms, emphasizing the critical importance of accurate and real-time monitoring of intracranial pressure to guide clinical treatment. This article reviews invasive intracranial pressure monitoring, which is widely utilized in clinical practice and has a high degree of accuracy.

### Literature search

PubMed, Medline, and Embase were utilized as search tools to identify relevant studies and articles, with the final search conducted on April 1st, 2023. The following search terms were employed: **“**intracerebral hemorrhage,” “cerebral hemorrhage,” “hypertensive intracerebral hemorrhage,” “hypertensive cerebral hemorrhage,” “intracranial pressure,” “ICP,” “intracranial pressure monitoring,” and “invasive intracranial pressure monitoring.” The exclusion criteria comprised all article types not written in the English language and/or without a full-text publication, single case reports, and those that included pediatric patients.

### Search results

**Figure 1:**
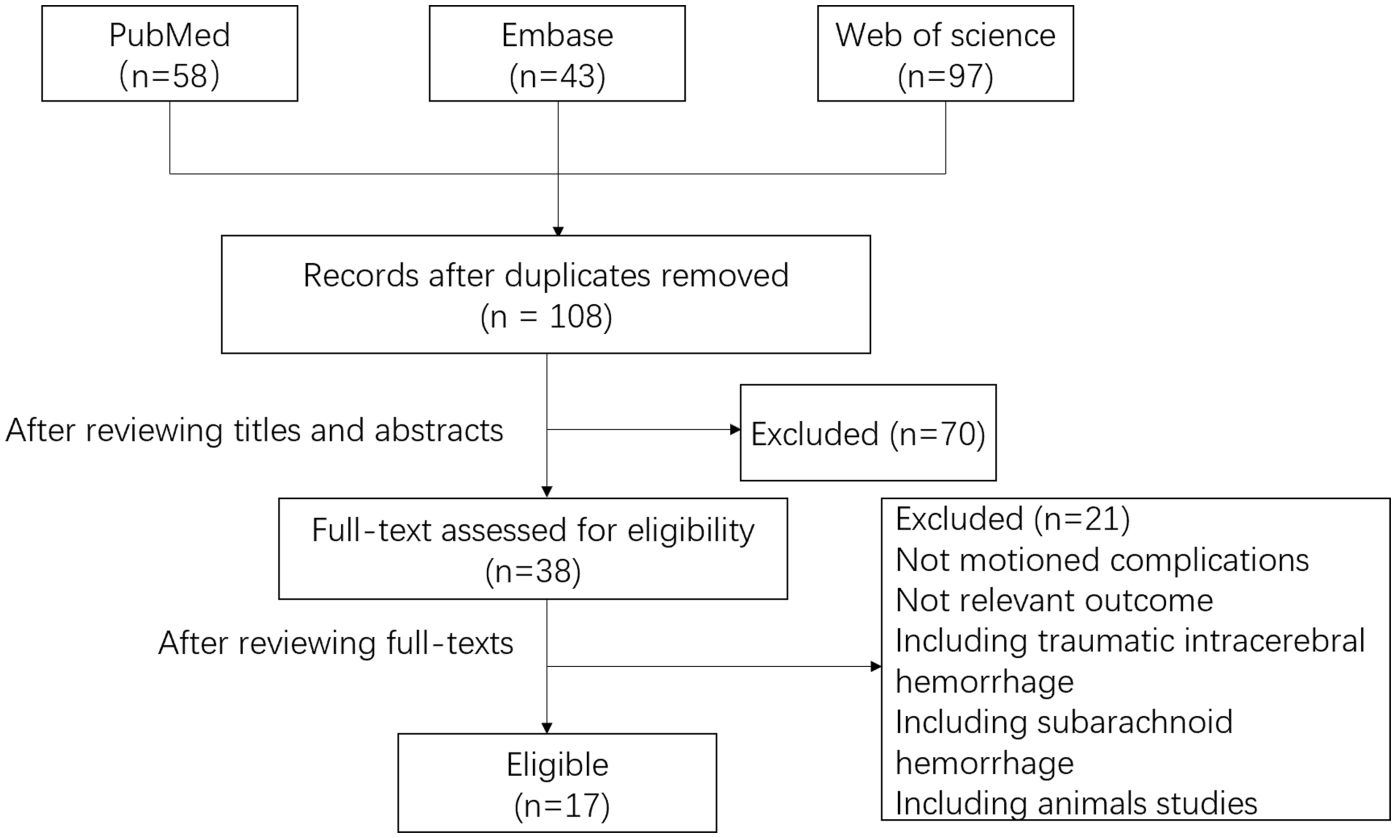
Flow chat of the study.

From the aforementioned search parameters, Figure 1 shows the study screening and selection process. The systematic database search yielded 58, 97, and 43 records from the PubMed, Web of Science, and Embase databases, respectively. After excluding duplicates and reviewing titles and abstracts, 38 articles were considered potential studies on invasive intracranial pressure monitoring following surgery for hypertensive cerebral hemorrhage. After reviewing the full-text, we have identified 17 primary studies that conform to the inclusion criteria and provide a comprehensive analysis of the monitoring techniques, monitoring duration, associated complications, and study conclusions. This data has been succinctly summarized in Table 1.

**Table 1:** Summary of invasive intracranial pressure monitoring.

Author	Study design/sample	Methods of monitoring	Complication	Mean monitoring time	Outcome
Dimitriou, et al.	Retrospective/288	External ventricular drainage Intraparenchymal monitors	Infection (9.2%) rebleeding (1.2%) infection (0.8%) rebleeding (0.8%)	3.9 days (with the greatest incidence of infections between day 5 and 11)	EVD is an indispensable device in neurosurgery. Unfortunately, it has a significantly high complication rate, mostly in relation to infections.
Yu, et al.	Retrospective/80	Invasive intracranial pressure monitoring	Encephaledema (68%) Cerebral infarction (12.5%) rebleeding (20%)	1 to 7 days after surgery	Guiding the clinical intervention actively to improve the surgery outcome.
Chen, et al.	Retrospective/3000	Invasive intracranial pressure monitoring	infection (6.4%)	No	Did not improved functional outcomes
Che, et al.	Retrospective/116	Invasive intracranial pressure monitoring	Infection (14%)	No	ICP implantation was associated with a better 6-month functional outcome.
Ren, et al.	Observational/196	Invasive intracranial pressure monitoring	Infection rate between ICP monitoring and no ICP monitoring did not differ significantly	The median duration of ICP monitoring was 6 days.	ICP monitoring is associated with a better 6-month functional outcome
Al-Kawaz, et al.	Post-hoc analysis, clinical trial/499	External ventricular drainage Intraparenchymal monitors	No	Median 3 days(1–6)	Decreasing high ICP and low CPP burden is associated with improved short- and long-term mortality.
Author	Study design/sample	Methods of monitoring	Complication	Mean monitoring time	Outcome
Men, et al.	Retrospective/150	Intraparenchymal monitors (IPMs)	Rebleeding (6%) infection (2%) Cerebral hernia (2%)	No	ICP monitors significantly improved clinical effect and treatment outcomes of HICH patients.
Menacho, et al.	Clinical trial/494	External ventricular drainage Intraparenchymal monitors (IPMs)	Intracranial infection (7.1%) Worsened or new intracranial hemorrhage (41.4%)	No	ICP monitors placed there is an association with poor neurologic outcome
Wang, et al.	Retrospective/106	External ventricular drainage Intraparenchymal monitors (IPMs)	Intracranial infection (0%) rebleeding (0%)	No	ICP monitors improve the prognosis of patients with HICH, reduce postoperative complications, and improve postoperative activities and quality of life.
Wu, et al.	Retrospective/53	External ventricular drainage Intraparenchymal monitors (IPMs)	Infection (0) rebleeding (0)	No	ICP monitors might improve the clinical effect and treatment outcomes of the patients with HICH.
Dallagiacoma, et al.	Observational/587	Invasive intracranial pressure monitoring	No	The median duration of ICP monitoring was 9 days	ICP monitoring was associated with a significant reduction of 6-month mortality，but not with neurologic outcome
Yang, et al.	Retrospective/53	Invasive ICP monitoring	No	No	The mean ICP is not the only indicator in HICH treatment，
Author	Study design/sample	Methods of monitoring	Complication	Mean monitoring time	Outcome
Kamel, et al.	Retrospective/243	Invasive intracranial pressure monitoring	No	No	Intracranial hypertension is common after ICH, especially in younger patients with supratentorial hemorrhage. Given active treatment of elevated ICP, intracranial hypertension does not appear associated with long-term outcomes, suggesting that ICP elevations should not necessarily be taken to signify a poor prognosis
Sykora, et al.	Retrospective/121	Intraparenchymal pressure monitoring external ventricular drainage	No	No	Intracranial pressure monitoring can improve mortality and poor outcome after ICH.
AUCOIN, et al.	Retrospective/350	Invasive intracranial pressure monitoring	Infection (7.9–21.9%)	No	No
North, et al.	Retrospective/378	Invasive intracranial pressure monitoring	Infection (0–3.6%)	Mean 45.6 h to 69.5 h	The subdural catheter and Richmond screw are safer than the ventricular catheter
Ehtisham, et al.	Retrospective/29	External ventricular drainage	Infection (0%)	No	placement of EVDs and ICP monitors by neurointensivists may be safe and effective

No indicates that this paper does not mention this content.

## The significance of intracranial pressure for the patient

Intracranial pressure refers to the force exerted by the contents of the cranial cavity on the surrounding lining. The cerebrospinal fluid plays a crucial role in maintaining a stable intracranial pressure, and any disruptions between its production and absorption can affect the pressure (10). For patients suffering from cerebral hemorrhage, analyzing and measuring intracranial pressure can be essential for determining the most effective therapeutic management measures (11). By measuring the intracranial pressure, the patient’s cerebral perfusion pressure can be estimated using the formula: cerebral perfusion pressure = mean arterial pressure−mean intracranial pressure. An increase in intracranial pressure may hinder blood flow, leading to ischemia, while an increase in cerebrovascular perfusion pressure may result in overperfusion, vasogenic edema, and secondary effects due to increased intracranial pressure (12). Intracranial hypertension is a significant cause of secondary brain injury and is also closely related to poor prognosis. Regular monitoring of intracranial pressure and cerebral perfusion pressure may therefore alter the prognosis of patients with brain injury (13–15). However, assessing changes in intracranial pressure and cerebral perfusion pressure from clinical observations of the patient’s state of consciousness and radiological changes can be challenging (8).

## Optimal duration of invasive intracranial pressure monitoring and the most common complications

There are no established guidelines or expert consensus regarding the optimal duration of intracranial pressure monitoring. A retrospective study conducted by Julien Dimitriou et al. revealed that the use of intracranial pressure monitoring for more than 5 days increased the risk of infection (16). It has also been suggested that the risk of infection associated with intracerebroventricular pressure monitoring increases after 5 days, with an overall infection rate of approximately 5%. However, the risk of infection with parenchymal monitoring is lower than that of intracerebroventricular monitoring (12). Therefore, the five-day mark may be an optimal time point for intracranial pressure monitor placement, but further studies are necessary to validate this conclusion. Infection is the most frequent complication associated with invasive intracranial pressure monitoring. In a study of 123 patients who underwent parenchymal intracranial pressure monitoring, only one case of infection was reported, compared to 16 out of 173 patients who underwent extra-ventricular drainage, and the duration of catheterization was found to be associated with the infection rate (16). Moreover, a meta-analysis indicated that the use of intracranial pressure monitoring was linked to a reduced rate of infection and an improved prognosis compared to the group without intracranial pressure monitoring (17). In a retrospective study of 213 patients with traumatic brain injury who had undergone invasive intracranial pressure monitoring, Alexander Bumberger et al. concluded that although there may be some complications associated with this method, they are acceptable. Furthermore, invasive intracranial pressure monitoring reduces the number of cranial CT scans performed on patients with increased intracranial pressure or those with a declining level of consciousness. This, in turn, lowers the number of patient evacuations and the hazards associated with transportation (18). A meta-analysis demonstrated that aggressive intracranial pressure monitoring and treatment resulted in a better prognosis for patients (19).

## The need for intracranial pressure monitoring in patients with hypertensive cerebral hemorrhage

Hypertensive cerebral hemorrhage results from the rupture of a blood vessel in the brain due to hypertension, leading to the accumulation of blood in brain tissue and the formation of a hematoma. This, in turn, causes distortion and deformation of the brain tissue (20). Regardless of whether conservative medication or surgery is used for treatment, hematoma formation is inevitable following cerebral hemorrhage. On the one hand, the hematoma compresses the healthy brain tissue surrounding it, leading to tissue death, while, on the other hand, the secondary damage caused by the mechanical effect of the expanding hematoma and the toxic effect of its degradation products on the surrounding brain tissue leads to the formation of peri-hematoma edema (21, 22). Furthermore, hypertensive cerebral hemorrhage usually affects deep brain structures such as the basal ganglia and thalamus (23). This poor functional prognosis is due to the presence of crucial neural nuclei in the deep brain. Hematomas after cerebral hemorrhage may compress the circulatory pathways of the cerebrospinal fluid, leading to obstructive hydrocephalus (24). The growth of the acute hematoma, the enlargement of the ventricles, and the edema of the tissue surrounding the hematoma all contribute to the deterioration of the patient’s neurological function (25). The occupying effect of the hematoma, progressive perihematomal edema, and hematoma widening can cause a reduction in cerebral perfusion pressure, an increase in intracranial pressure, and even cerebral herniation (26). Recurrence of hemorrhage, edema and hydrocephalus after hypertensive cerebral hemorrhage can all causes an increase in intracranial pressure and a deterioration in the patient’s level of consciousness. However, changes in intracranial pressure occur prior to the onset of clinical signs and symptoms in patients. For patients undergoing intracranial pressure monitoring, a quick review of cranial Computed Tomography can be performed at the time of intracranial pressure elevation so that patients can receive treatment before their condition worsens.

## Methods of invasive intracranial pressure monitoring

In current clinical practice, methods for measuring intracranial pressure include both invasive and non-invasive techniques. Although non-invasive intracranial pressure monitoring has the advantage of not causing trauma or complications to patients due to the placement of monitor sensors, its inaccuracy and unreliability have limited its widespread use (27). The potential for inaccurate intracranial pressure readings to mask a patient’s critical condition and mislead clinicians underscores the importance of using invasive intracranial pressure monitoring as the main method for dynamic, real-time clinical monitoring of intracranial pressure changes. Despite the availability of various non-invasive intracranial pressure measurement methods, none of them can provide the same level of accuracy as invasive intracranial pressure monitoring (28). Therefore, invasive intracranial pressure monitoring continues to be the preferred method, requiring the insertion of a sensor probe into the brain parenchyma or ventricles using a cranial drill.

Intracerebroventricular pressure monitoring: invasive intracranial pressure monitoring, combined with extra-ventricular drainage, is considered the gold standard for monitoring intracranial pressure (29, 30). This technique involves inserting a catheter into one of the lateral ventricles, with the external transducer placed at the same level as the Monro foramen (31). This method has various advantages. Firstly, the measurement of pressure can be used as a therapeutic tool by opening the drainage tube when the intracranial pressure is elevated. Secondly, the intracerebroventricular pressure monitoring catheter and the ICP probe share the same device, meaning that even if the catheter becomes blocked, the pressure can still be measured normally (30). Furthermore, the intracerebroventricular pressure monitoring method has the ability to be zeroed *in vivo* (32). However, there are limitations to this technique. For instance, in cases of congenital small ventricles or where the ventricles are deformed by pressure due to severe cerebral edema, the placement of an external ventricular drainage can be technically challenging (27). In addition, this method is mainly prone to high infection rates and is not suitable for long-term monitoring as data show that the risk of intracranial infection begins to increase five days after the placement of an extra-ventricular drain (27, 29). Of the 155 patients who received extra-ventricular drainage included by Fabrizio Ortolano’study, 33 experienced minor bleeding associated with extra-ventricular drain placement, but none of these cases caused deterioration in neurological function or required surgical intervention to remove the hematoma (33).

Intracerebral parenchymal pressure monitoring: In patients who undergo surgical treatment for cerebral hemorrhage, whether through minimally invasive drilling and drainage or decompressive craniectomy, intracerebral parenchymal pressure measurement necessitates the placement of transducers without additional boreholes, allowing the transducer probe to be placed using surgical access to avoid unnecessary patient harm. However, there is a zero point drift, and the parenchymal probe cannot be recalibrated after placement (27). Studies on traumatic brain injury have shown that parenchymal intracranial pressure monitoring is equally accurate as intracerebroventricular pressure measurement, and that intracerebroventricular pressure measurement results in a worse prognosis and higher mortality compared to intracerebroventricular pressure measurement devices, with one of the drawbacks being the inability to drain cerebrospinal fluid (34). Moreover, intracranial pressure measured by ventricular drains is considered the gold standard for measuring overall pressure, whereas for intracerebral parenchymal pressure monitoring, when there is an intracranial pressure gradient, the measurement cannot substitute for the overall intracranial pressure (35).

The intracranial pressure monitoring methods of epidural and subdural monitoring are infrequently used in clinical practice due to their low accuracy and unreliability (36, 37). In contrast, non-invasive methods of intracranial pressure monitoring have gained popularity due to their practicality, reproducibility, safety, and lack of known side effects. An increase in intracranial pressure can cause the optic nerve sheath diameter in the retrobulbar chamber to expand, and ultrasound can be used as a non-invasive tool for assessing intracranial pressure by measuring the optic nerve sheath diameter (38). However, ocular ultrasound lacks a clear- cut-off value as an indicator of elevated intracranial pressure when using the optic nerve sheath diameter as an indirect assessment (39), and it is a difficult test that requires a high level of knowledge and a trained operator, which is one of its drawbacks (40, 41).

## Discussion

Monitoring intracranial pressure provides a crucial reference point for diagnosing and treating neurosurgical diseases, as well as determining intracranial diseases (42), Increased intracranial pressure is a frequent complication of cerebral hemorrhage and can be attributed to various factors such as the hematoma’s occupying effect, peri-hematoma edema, hematoma enlargement, altered cerebrospinal fluid hemodynamics, and subsequent hydrocephalus formation. Intracranial hypertension is independently related to clinical prognosis and patient mortality (43–45). Daniel Agustín Godoy et al.’s meta-analysis revealed that up to 67% of patients had intracranial hypertension after cerebral hemorrhage. This figure was obtained after excluding patients who had abandoned further treatment or had been externally drained from the ventricles. The mortality rate associated with increased intracranial pressure was 50% in four studies involving 239 patients (45). The value of intracranial pressure monitoring lies in its ability to intervene before irreversible brain damage occurs, thus allowing clinicians to make informed treatment decisions (46).

Published studies have largely demonstrated that intracranial pressure monitoring has a positive effect on patient prognosis. In a retrospective study involving 196 patients, Junwei Ren et al. concluded that patients who received intracranial pressure monitoring had better clinical outcomes and lower mortality rates at 6 months post-discharge (44). However, a retrospective study by Che et al. (46) found that invasive intracranial pressure monitoring did not prolong hospital stays, reduce patient mortality, or improve functional recovery and prognosis 6 months after hypertensive cerebral hemorrhage. Furthermore, some patients may be treated using less invasive methods with the assistance of intracranial pressure monitoring (47). On the one hand, this method avoids unnecessary major trauma for the patient, and on the other hand, having a reference for intracranial pressure values provides assurance for the clinician’s management. Additionally, monitoring and normalizing intracranial pressure can reduce secondary neurological injury and its associated morbidity and mortality (48). These facts highlight the value of intracranial pressure monitoring after cerebral hemorrhage, which is often considered life-saving (31). In clinical practice, patients’ intracranial pressure values often precedes changes in clinical symptoms and signs (49). Moreover, early symptoms may be atypical and overlooked, but once the obvious symptoms appear, the patient’s optimal window of treatment may be have passed.

## Conclusion

Currently, invasive intracranial pressure monitoring is deemed the most dependable method for monitoring intracranial pressure in real-time and with precision. However, further prospective, multi-center and multi-data research is still necessary to fully comprehend its impact on patient outcomes. As society develops, there is a growing demand for minimally invasive or even non-invasive techniques. With advancements in technology, non-invasive intracranial pressure monitoring is expected to become the primary method for monitoring intracranial pressure in the future, provided that technical challenges related to accuracy, stability, and real-time monitoring are overcome. As such, non-invasive intracranial pressure monitoring is currently an active area of research.

## Author contributions

All authors listed have made a substantial, direct, and intellectual contribution to the work and approved it for publication.

## Conflict of interest

The authors declare that the research was conducted in the absence of any commercial or financial relationships that could be construed as a potential conflict of interest.

## Publisher’s note

All claims expressed in this article are solely those of the authors and do not necessarily represent those of their affiliated organizations, or those of the publisher, the editors and the reviewers. Any product that may be evaluated in this article, or claim that may be made by its manufacturer, is not guaranteed or endorsed by the publisher.
